# Cardiometabolic disorder reduces survival prospects more than suboptimal body mass index irrespective of age or gender: a longitudinal study of 377,929 adults in Taiwan

**DOI:** 10.1186/s12889-018-5038-0

**Published:** 2018-02-14

**Authors:** Chih-Cheng Hsu, Mark L. Wahlqvist, I-Chien Wu, Yu-Hung Chang, I-Shou Chang, Yi-Fen Tsai, Ting-Ting Liu, Chwen Keng Tsao, Chao A. Hsiung

**Affiliations:** 1 Institute of Population Health Sciences, National Health Research Institutes, 35 Keyan Road, Zhunan, Miaoli County, Taiwan; 2 Department of Health Services Administration, China Medical University, Taichung, Taiwan; 3 Department of Family Medicine, Min-Sheng General Hospital, Taoyuan, Taiwan; 4 Department of Epidemiology and Preventive Medicine, Monash University, Melbourne, Australia; 5 Department of Public Health, China Medical University, Taichung, Taiwan; 6 National Institute of Cancer Research, National Health Research Institutes, Zhunan, Taiwan; 7 MJ Health Management Institution, Taipei, Taiwan

**Keywords:** Cardiometabolic disorder, Blood pressure, Glucose, Diabetes, BMI, Underweight, Obesity, Elderly, Mortality

## Abstract

**Background:**

The effect of cardio-metabolic profile on the relationship of body mass index (BMI) with mortality is unclear. The aim of this study was to explore association between BMI and mortality at all ages, taking account of cardio-metabolic disorders.

**Methods:**

We followed 377,929 individuals (≥ 20 years), who registered for health checkups in 1996–2007, until 2008 and found 9490 deaths. From multivariable Cox proportional hazards models we estimated mortality hazard ratios (HR) for those in high blood pressure, hyperglycemia, high waist circumference, dyslipidemia, and different BMIs categories (the underweight [< 18.5 kg/m^2^], low normal weight [18.5–21.9 kg/m^2^], normal weight [22–23.9 kg/m^2^, the referent], overweight [24–26.9 kg/m^2^], obese1 [27–29.9 kg/m^2^], and obese2 [≥ 30 kg/m^2^]). Population attributable risk (PAR) provided estimates of the population mortality burden attributable to high blood pressure, hyperglycemia, high waist circumference, dyslipidemia, and deviant BMIs.

**Results:**

Higher blood pressure, hyperglycemia, high waist circumference, and dyslipidemia were significantly predictive of higher mortality for nearly all ages. Compared with the referent BMI, underweight (HR = 1.69, 95% confidence interval = 1.51–1.90) and low normal weight (HR = 1.19, 1.11–1.28) were significant mortality risks, while overweight (HR = 0.82, 0.76–0.89) and obese1 (HR = 0.88, 0.79–0.97) were protective against premature death. The mortality impact of obesity was largely attributable to cardio-metabolic profile and attenuated by age. The population mortality burden with high blood pressure (PAR = 7.29%), hyperglycemia (PAR = 5.15%), high waist circumference (PAR = 4.24%), and dyslipidemia (PAR = 5.66%) was similar to that in the underweight (PAR = 5.50%) or low normal weight (PAR = 6.04%) groups. Findings for non-smokers and by gender were similar.

**Conclusions:**

The effect of BMI on mortality varies with age and is affected by cardio-metabolic status. Compared to any deviant BMI, abnormal cardio-metabolic status has a similar or even greater health impact at both the individual and population levels.

**Electronic supplementary material:**

The online version of this article (10.1186/s12889-018-5038-0) contains supplementary material, which is available to authorized users.

## Background

There is a general understanding that the body mass index (BMI) in itself is a major determinant of mortality [1], although qualified by fat distribution for disease-specific outcomes [2]. Historically, this may not always have been so [3]; but evidence from large-scale cohorts in both Western and Asian countries generally indicate that a BMI of 22–25 kg/m^2^ may be optimal for prevention of premature death [1, 4–9]. While underweight (BMI < 18.5 kg/m^2^) is usually recognized as a health hazard, especially for the elderly [10, 11], the mortality risk of a high BMI is probably attenuated by age [4, 5, 12–16]. However, to what extent the association between BMI and mortality is altered by cardio-metabolic factors (CMFs) is unclear.

Apart from their apparent cardiovascular risks, metabolic syndrome (MetS) and insulin resistance could aggravate mortality risk in any BMI category [17]. The Emerging Risk Factors Collaboration [18] has demonstrated a reduced effect of obesity on the risk of coronary heart disease and ischemic stroke by adjustment for CMFs such as systolic blood pressure, history of diabetes, and lipid profiles. Presumptively, but not necessarily, the principal underlying disorder here is one of energy regulation manifest in a body compositional disorder, with CMFs acting in concert with or independently of BMI to determine mortality risk [19]. Few studies have been designed to evaluate the relative importance of CMFs. For example, in a meta-analysis [20], Flegal and colleagues concluded that obesity (BMI ≥ 30 kg/m^2^) increases all-cause mortality while overweight (BMI = 25–30 kg/m^2^) reduces it; however, age was not stratified, nor was cardio-metabolic risk taken into account. In an era when the prevalence of MetS has sharply increased [21], the predictive power of BMI for mortality relative to that of cardio-metabolic risk factors, particularly hypertension and hyperglycemia, is required.

For these reasons, we have studied a large population cohort of adult Taiwanese for up to 13 years to determine the mortality risk for BMI across a wide range and, at the same time, the mortality risks of CMFs so that we could establish their relative population attributable risk (PAR).

## Methods

### Participants

In this prospective cohort study, the participants came from the MJ Health Screening Center in Taiwan. The center’s operations have been described elsewhere [22]. Briefly, 473,543 individual members were enrolled during 1996–2007 for health checkups at least once and followed to the end of 2008. Of these enrollees, 19,634 younger than 20 years (4.1%); 20,491 with self-reported comorbidities of cancer, heart disease, or stroke at entry (4.3%); and 55,489 with incomplete anthropometric or laboratory data (11.7%) were excluded. The remaining 377,929 participants were studied. The mean follow-up time for this selected cohort was 8.1 years.

### Measurements

#### Body mass index and its classification

Anthropometric indicators comprised height and weight measurements. The BMI was calculated by dividing weight (in kg) by the square of height (in meters). We primarily categorized BMI into four groups: underweight (<18.5 kg/m^2^), normal weight (18.5–23.9 kg/m^2^), overweight (24–26.9 kg/m^2^), and obese (≥ 27 kg/m^2^), according to the definitions of the Department of Health in Taiwan [23]. Because the mortality risk might be lowest for people with a BMI of 22.5 to 25 [1, 4–9], we further subdivided the “normal” BMI category (which contained a majority of 55.9% of the participants) into “low normal” (18.5–21.9 kg/m^2^), and “normal” (22–23.9 kg/m^2^) categories. Likewise, for the obesity group, the subsets of obese2 (≥ 30 kg/m^2^) and obese1 (27–29.9 kg/m^2^) were generated to acknowledge the WHO definition of obesity as a BMI ≥ 30 kg/m^2^ [24].

#### Metabolic syndrome (MetS)

Overnight fasting blood was collected at entry and analyzed for plasma glucose, triglyceride, and HDL cholesterol using an auto-analyzer (Hitachi 7150, Tokyo, Japan). The modified NCEP/ATP III criteria were used for the diagnosis of the metabolic syndrome. A person who had three or more of the following risk components was considered to have MetS: waist circumference ≥ 90 cm in men or ≥80 cm in women, triglyceride ≥150 mg/dL or on anti-hyperlipidemia medication, HDL cholesterol <40 mg/dL in men or <50 mg/dL in women, blood pressure ≥ 130/85 mmHg or on anti-hypertension medication, and fasting glucose >100 mg/dL or on anti-diabetes medication. In previous studies, the modified ATP III criteria have been shown to be predictive of mortality and cardiovascular diseases in Asians including Taiwanese [25].

#### Death ascertainment

The cohort database was linked to the electronic national death records between 1996 and 2008 by use of identification numbers (ID) but with a scrambling protocol to preserve anonymity. There were 9490 deaths identified during the follow-up period. Where date of death was not ascertainable in this way, we assumed participants were alive from the entry date until 31 December 2008.

#### Covariates

In addition to BMI and metabolic parameters, other covariates for analysis included age, gender, education level, and the personal behaviors of smoking, alcohol drinking, and leisure time physical activity (LTPA). Baseline age was established from the enrollees’ birth date and the date of the first health examination. We categorized participants into five age groups: 20–39, 40–49, 50–59, 60–69, and 70 years and over. Education was specified by school years and dichotomized as ≤6 years and >6 years. Current smokers were those who smoked at least once a week; otherwise, subjects were defined as non-smokers. Current drinkers were those who drank an alcoholic beverage at least once a week. LTPA was dichotomized by frequency of engagement: “none or less than 1 hour per week” and “at least 1 hour per week.”

#### Statistical analysis

Mean and standard deviation (SD) were used to describe continuous and percentages for categorical variables. Comparison of differences in means and percentages were performed using Student’s t test and the chi-square test, respectively. For comparative mortality risk with BMI, we stratified participants into the 6 BMI groups by age as indicated. The mortality rate in the follow-up period was expressed as the number of deaths per 10,000 person years. The person-years were calculated as the time elapsed from the entry date until the date of death, or the end of follow-up, whichever came first. The calculation of a 95% confidence interval (CI) for the mortality rate was based on the assumption that the number of deaths followed a Poisson distribution. We estimated the mortality rate in each BMI-age subgroup and with a further stratification by MetS status. We also calculated the mortality rate ratio (RR) to compare the mortality rate for individuals with MetS to those without MetS, in each BMI-age subgroup.

Multivariable Cox proportional hazards models were used to explore mortality risks of different BMIs, compared to the referent “normal” BMI (22–23.9 kg/m^2^). Study entry was defined as the date of enrollment. Observations were censored at the end of the study or the date that individuals died, whichever occurred first. Results were expressed as hazard ratios (HRs) and 95% CIs compared with the referent groups. To eliminate survival bias, we performed sensitivity analyses by exclusion of those who died within 1, 2, or 3 years after entry. Multivariable Cox regression analyses were also separately conducted for non-smokers, men, and women for the subgroup analysis. The proportional hazards assumption was evaluated by comparing estimated log-log survival curves for all covariates in each analysis. All assessed log-log survival plots graphically showed two parallel lines, indicating no violation of the assumption. In addition, we assessed the proportion of disease burden in Taiwan attributable to different BMI, hypertension, and hyperglycemia for people at different age groups by calculating their population attributable risk (PAR) [26]. Analyses were performed with SAS software, version 9.1 (SAS Institute, Cary, NC). A two-sided *P* value of 0.05 was considered statistically significant.

## Results

The crude mortality rate of study subjects in different age-BMI matches is shown in Table 1. For those younger than 50, overweight and obesity are associated with higher death rates; however, for people older than 50, those in the overweight category have the lowest mortality rate and those who are underweight have the highest. People with MetS generally have moderately higher mortality rates (mortality rate ratio (RR) = 1.2–2.8), irrespective of age-BMI category — except for those in the group with BMI ≥ 30 kg/m^2^ (RR inconsistent) or in the underweight younger groups (< 50 years, RR = 5.5–11.6), who have much higher mortality rates.

**Table 1:** Mortality rate (per 10,000 person-years) by body mass index, age, and status of metabolic syndrome

	Underweight				Low normal				Normal				Overweight				Obese1				Obese2			
	No. of deaths	Person years (× 10^3^)	Death rate	RR	No. of deaths	Person years (× 10^3^)	Death rate	RR	No. of deaths	Person years (× 10^3^)	Death rate	RR	No. of deaths	Person years (×10^3^)	Death rate	RR	No. of deaths	Person years (× 10^3^)	Death rate	RR	No. of deaths	Person years (× 10^3^)	Death rate	RR
Overall																								
**20–39**	93	201.9	**4.6**		313	683.9	**4.6**		180	324.3	5.6		170	276.9	6.1		84	97.4	8.6		67	43.4	15.4	
**40–49**	37	18.7	19.8		215	159.4	**13.5**		205	143.3	14.3		267	162.5	16.4		127	62.0	20.4		68	23.4	29.1	
**50–59**	70	9.5	73.7		420	94.2	44.6		474	114.6	41.4		589	147.9	**39.8**		300	61.5	48.8		116	22.0	52.7	
**60–69**	165	8.7	189.7		729	59.7	122.1		741	69.2	107.1		916	91.0	**100.7**		408	36.8	100.9		157	12.7	123.6	
**≥ 70**	222	4.5	493.3		715	20.4	350.5		560	20.2	277.2		702	26.6	**263.9**		292	10.5	278.1		98	3.4	288.2	
With MetS																								
**20–39**	4	0.8	50.0	11.6 (0.2–21.9)	11	10.9	10.1	2.3 (0.9–3.6)	15	19.2	**7**.**8**	1.5 (0.7–2.3)	44	45.1	9.8	1.8 (1.3–2.4)	37	36.5	10.1	1.5 (1.0–2.0)	42	24.9	16.9	1.4 (1.0–1.9)
**40–49**	3	0.3	100.0	5.5 (0–12.8)	25	7.2	34.7	2.8 (1.7–3.9)	33	16.7	**19**.**8**	1.5 (1.0–2.0)	91	43.2	21.1	1.6 (1.2–1.9)	77	30.0	25.7	1.5 (1.2–1.8)	45	15.0	30.0	1.4 (1.0–1.8)
**50–59**	3	0.3	100.0	1.5 (0–3.2)	76	10.0	76.0	1.9 (1.5–2.4)	138	24.7	55.9	1.6 (1.3–1.9)	258	54.7	**47**.**2**	1.5 (1.3–1.6)	196	35.5	55.2	1.5 (1.3–1.8)	74	14.7	50.3	0.9 (0.7–1.1)
**60–69**	16	0.6	266.7	1.5 (0.8–2.3)	166	10.6	156.6	1.4 (1.2–1.6)	266	21.3	124.9	1.3 (1.1–1.5)	478	44.4	**107**.**7**	1.2 (1.1–1.3)	286	24.1	118.7	1.2 (1.1–1.4)	119	9.4	126.6	1.3 (1.0–1.5)
**≥ 70**	33	0.4	825.0	1.9 (1.4–2.8)	219	4.9	446.9	1.5 (1.3–1.7)	246	8.2	300.0	1.2 (1.1–1.4)	416	14.9	**279**.**2**	1.3 (1.2–1.5)	208	7.3	284.9	1.7 (1.4–1.9)	75	2.5	300.0	0.9 (0.7–1.1)
Without MetS																								
**20–39**	86	198.4	**4**.**3**		289	653.1	4.4		150	288.4	5.2		112	211.8	5.3		35	52.7	6.6		18	15.2	11.8	
**40–49**	33	18.0	18.3		182	145.9	**12**.**5**		158	117.3	13.5		141	103.8	13.6		45	26.0	17.3		13	6.0	21.7	
**50–59**	60	8.8	68.2		313	78.7	39.8		281	80.3	35.0		255	79.0	**32**.**3**		72	20.1	35.8		29	5.1	56.9	
**60–69**	135	7.5	180.0		488	44.5	109.7		394	41.1	95.9		327	37.1	**88**.**1**		90	9.3	96.8		22	2.2	100.0	
**≥ 70**	169	3.8	444.7		409	13.9	294.2		245	10.1	242.6		190	9.0	211.1		35	2.1	**166**.**7**		14	0.4	350.0	

MetS: metabolic syndrome

RR: mortality rate ratio, comparing the mortality rate for individuals with MetS to those without MetS, with regard to the corresponding BMI-age subgroups

The numbers in bold indicate the lowest death rate in the respective age groups

**Fig. 1:**
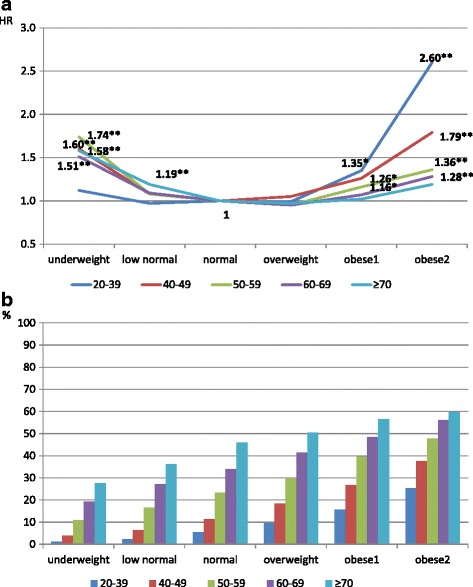
All-cause mortality risk (**1a**) and proportion of high blood pressure and hyperglycemia (**1b**) in different BMI categories for overall study subjects stratified by age. The hazards ratios shown in 1A were derived from Cox proportional hazards models adjusted for gender, age, education level, smoking status, physical activity, and drinking status. *P < 0.05, **P < 0.01

**Fig. 2:**
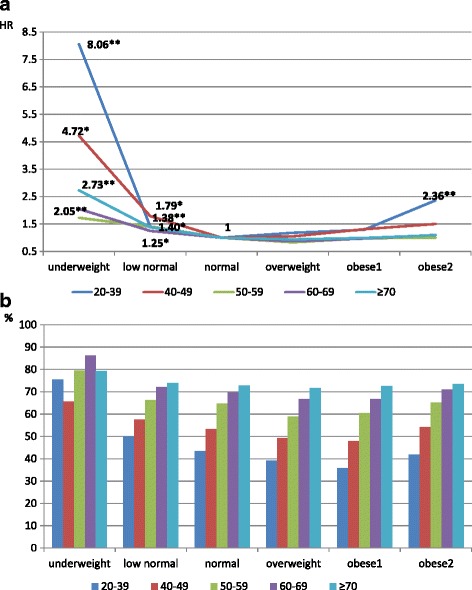
All-cause mortality risk (**2a**) and proportion of subjects with both high blood pressure and hyperglycemia (**2b**) in different BMI categories for subjects with the metabolic syndrome, stratified by age. The hazard ratios shown in 2A were derived from Cox proportional hazards models adjusted for gender, age, education level, smoking status, physical activity, and drinking status. *P < 0.05, **P < 0.01

The multivariable adjusted hazard ratios (HR) are shown in Fig. 1 (for subjects overall) and 2 (for those with MetS). Using those with BMI = 22–23.9 kg/m^2^ as referent, the obese and underweight were more likely to die; the mortality risk of obesity is even more apparent in the younger groups (Fig. 1a). Figure 1b shows an increasing proportion with both high blood pressure and hyperglycemia with increasing BMI at all ages. However, for those with MetS, the mortality risk of being underweight is much greater than that of obesity, especially for younger people (Fig. 2a). Figure 2b reveals a high proportion with both high blood pressure and hyperglycemia in each age-BMI category, particularly and homogeneously in the underweight groups (65–85%), but with more of a gradient by age in the higher BMI categories. This indicates that the high mortality in underweight groups who have MetS is predominantly related to CMFs.

**Fig. 3:**
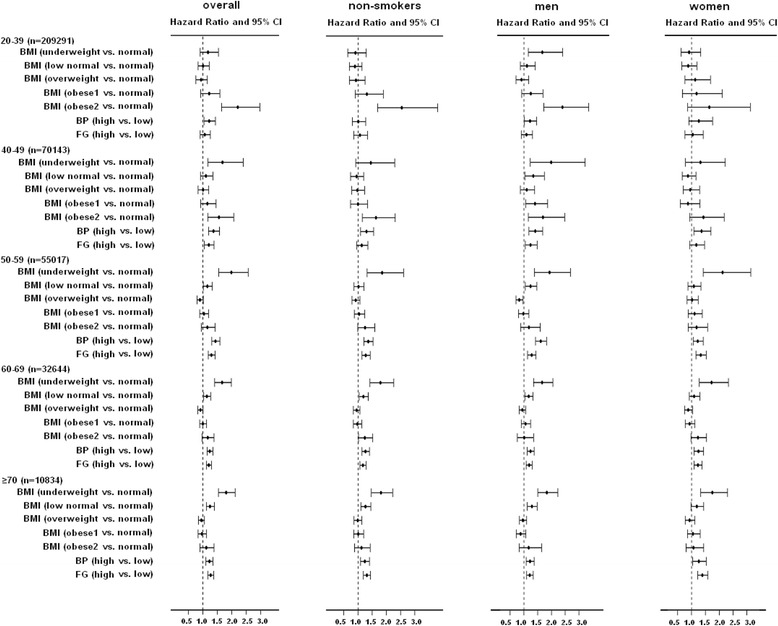
Adjusted mortality risks for different BMI, high blood pressure, and hyperglycemia categories for overall subjects, non-smokers, men, and women — each stratified by age. BMI classification: underweight: <18.5 kg/m^2^, low normal: 18.5–21.9 kg/m^2^, normal: 22–23.9 kg/m^2^, overweight: 24–26.9 kg/m^2^, obese1: 27–29.9 kg/m^2^, obese2: ≥ 30 kg/m^2^. The hazards ratios shown in Fig. 3 were derived from Cox proportional hazards models adjusted for gender, age, education level, smoking status, physical activity, and drinking status. The *P* value for interaction between BMI and sex is 0.3168, 0.6538, 0.0520, 0.1910, and 0.0387 for age group 20–39, 40–49, 50–59, 60–69, and ≥70, respectively

Figure 3 shows the subgroup analyses, indicating a similar mortality pattern among the different age-BMI groups for subjects overall, as for the subgroups of non-smokers, whether men or women. The mortality risks of high blood pressure and hyperglycemia were both significant for those older than 50 in subjects overall, as well as in each subgroup. In order to eliminate indication bias, we conducted sensitivity analyses by deleting those who died within 1, 2, or 3 years, and we obtained similar findings (shown in Additional file 1: Table S1). The results were also robust in the models additionally adjusted for waist circumference (Additional file 1: Figure S1). In another subgroup analysis, we added those who had self-reported heart disease or stroke into our study subjects (*N* = 390,941) to assess the robustness of the relationship between metabolic risk factors and mortality. As shown in Additional file 1: Table S2, the results were similar.

The comparison of the PAR for mortality between different BMI, high blood pressure, hyperglycemia, central obesity (higher waist circumference), and dyslipidemia is shown in Table 2. For subjects overall, the PARs for underweight, low normal weight, and obese2 were 5.5%, 6.0%, and 0.4%, respectively, all of which were lower than the PARs for high blood pressure (7.3%). Except for those ≥70 (with high PARs for underweight and low normal weight: 5.1% and 7.2%, respectively), the population mortality burdens of high blood pressure, hyperglycemia, central obesity, and dyslipidemia were generally higher than that caused by deviant BMI categories, related to the high prevalence of cardio-metabolic disorders in all age groups.

**Table 2:** Mortality risk, prevalence, and population attributable burden of mortality for different BMI, high blood pressure, and hyperglycemia in overall subjects and people in different age groups

	Model 1 HR	Model 2 HR	Model 3 HR	95% CI	Prevalence (%)	PAR (%)
Overall (*n* = 377,929, death = 9490)						
Underweight (vs. normal BMI)	0.77***	1.54***	1.69***	(1.51–1.90)	8.43	5.50
Low normal (vs. normal BMI)	0.74***	1.11***	1.19***	(1.11–1.28)	33.80	6.04
Normal BMI	1	1	1	–	22.10	–
Overweight (vs. normal BMI)	1.17***	0.97	0.82***	(0.76–0.89)	23.17	−4.35
Obese1 (vs. normal BMI)	1.39***	1.12**	0.88**	(0.79–0.97)	8.89	−1.08
Obese2 (vs. normal BMI)	1.52***	1.45***	1.12	(0.98–1.28)	3.61	0.43
High BP (vs. normal BP)	3.86***	1.32***	1.26***	(1.19–1.34)	30.22	7.29
High FG (vs. normal FG)	2.57***	1.25***	1.18***	(1.12–1.24)	30.17	5.15
High WC (vs. normal WC)	2.50***	1.13***	1.24***	(1.15–1.33)	18.47	4.24
Dyslipidemia vs. normal TG/HDL	1.36***	1.12***	1.14***	(1.08–1.20)	42.89	5.66
20–39 (*n* = 209,291, death = 907)						
Underweight (vs. normal BMI)	0.85	1.12	0.94	(0.68–1.30)	12.67	−0.77
Low normal (vs. normal BMI)	0.82*	0.97	1.00	(0.80–1.25)	41.22	0.00
Normal BMI	1	1	1	–	19.80	–
Overweight (vs. normal BMI)	1.11	0.99	0.85	(0.65–1.10)	17.19	−2.65
Obese1 (vs. normal BMI)	1.57***	1.35*	1.00	(0.69–1.47)	6.21	0.00
Obese2 (vs. normal BMI)	2.87***	2.60***	1.65*	(1.04–2.60)	2.92	1.86
High BP (vs. normal BP)	1.57***	1.37***	1.17	(0.97–1.43)	16.85	2.78
High FG (vs. normal FG)	1.42***	1.18*	1.10	(0.91–1.34)	19.15	1.88
High WC (vs. normal WC)	2.10***	1.70***	1.33	(0.97–1.82)	10.20	3.26
Dyslipidemia vs. normal TG/HDL	1.38***	1.23**	1.19*	(1.01–1.41)	37.91	6.72
40–49 (*n* = 70,143, death = 919)						
Underweight (vs. normal BMI)	1.44*	1.60**	1.66*	(1.10–2.50)	3.57	2.30
Low normal (vs. normal BMI)	0.96	1.08	1.15	(0.91–1.44)	28.60	4.11
Normal BMI	1	1	1	–	24.81	–
Overweight (vs. normal BMI)	1.15	1.05	0.92	(0.73–1.17)	28.16	−2.30
Obese1 (vs. normal BMI)	1.43**	1.26*	1.11	(0.80–1.53)	10.80	1.17
Obese2 (vs. normal BMI)	2.03***	1.79***	1.43	(0.95–2.16)	4.07	1.72
High BP (vs. normal BP)	1.61***	1.43***	1.28**	(1.08–1.51)	30.47	7.86
High FG (vs. normal FG)	1.44***	1.27***	1.12	(0.95–1.33)	35.44	4.08
High WC (vs. normal WC)	1.43***	1.22*	1.11	(0.86–1.42)	20.45	2.20
Dyslipidemia vs. normal TG/HDL	1.17*	1.04	1.00	(0.84–1.18)	46.82	0.00
50–59 (*n* = 55,017, death = 1969)						
Underweight (vs. normal BMI)	1.83***	1.74***	1.77***	(1.28–2.43)	2.21	1.67
Low normal (vs. normal BMI)	1.09	1.08	1.19*	(1.01–1.40)	21.36	3.90
Normal BMI	1	1	1	–	25.45	–
Overweight (vs. normal BMI)	0.96	0.95	0.74***	(0.63–0.87)	32.58	−9.26
Obese1 (vs. normal BMI)	1.17*	1.16*	0.76*	(0.62–0.95)	13.48	−3.34
Obese2 (vs. normal BMI)	1.28*	1.36**	0.88	(0.66–1.17)	4.92	−0.59
High BP (vs. normal BP)	1.46***	1.46***	1.48***	(1.32–1.67)	50.32	19.45
High FG (vs. normal FG)	1.39***	1.35***	1.17**	(1.05–1.31)	46.95	7.39
High WC (vs. normal WC)	1.23***	1.23***	1.39***	(1.19–1.62)	31.63	10.98
Dyslipidemia vs. normal TG/HDL	1.14**	1.09	1.09	(0.97–1.23)	49.40	4.26
60–69 (*n* = 32,644, death = 3116)						
Underweight (vs. normal BMI)	1.77***	1.51***	1.74***	(1.41–2.14)	3.14	2.27
Low normal (vs. normal BMI)	1.14*	1.09	1.14	(1.00–1.29)	21.59	2.93
Normal BMI	1	1	1	–	24.97	–
Overweight (vs. normal BMI)	0.94	0.95	0.82**	(0.72–0.93)	32.45	−6.20
Obese1 (vs. normal BMI)	1.03	1.07	0.85	(0.72–1.02)	13.15	−2.01
Obese2 (vs. normal BMI)	1.16	1.28**	1.00	(0.79–1.26)	4.69	0.00
High BP (vs. normal BP)	1.19***	1.24***	1.22***	(1.10–1.34)	65.80	12.65
High FG (vs. normal FG)	1.19***	1.22***	1.16**	(1.06–1.27)	52.60	7.76
High WC (vs. normal WC)	0.95	1.05	1.17*	(1.04–1.33)	39.28	6.26
Dyslipidemia vs. normal TG/HDL	1.12**	1.15***	1.14**	(1.04–1.25)	52.32	6.82
≥70 (*n* = 10,834, death = 2579)						
Underweight (vs. normal BMI)	1.80***	1.58***	1.96***	(1.61–2.39)	5.63	5.13
Low normal (vs. normal BMI)	1.27***	1.19**	1.32***	(1.15–1.52)	24.19	7.19
Normal BMI	1	1	1	–	23.47	–
Overweight (vs. normal BMI)	0.95	0.97	0.87	(0.75–1.01)	30.64	−4.15
Obese1 (vs. normal BMI)	0.96	1.02	0.90	(0.74–1.10)	12.13	−1.23
Obese2 (vs. normal BMI)	1.05	1.19	1.18	(0.90–1.54)	3.93	0.70
High BP (vs. normal BP)	1.16**	1.20***	1.13*	(1.01–1.28)	77.69	9.17
High FG (vs. normal FG)	1.22***	1.25***	1.26***	(1.14–1.39)	56.11	12.73
High WC (vs. normal WC)	0.91	1.02	1.21**	(1.06–1.39)	45.31	8.69
Dyslipidemia vs. normal TG/HDL	1.05	1.09*	1.19***	(1.07–1.32)	51.98	8.99

*P < 0.05, **P < 0.01, ***P < 0.001

BMI: body mass index, BP: blood pressure, FG: fasting glucose level, WC: waist circumference, TG: triglyceride, HDL: high density lipoprotein cholesterol

BMI classification: Underweight: <18.5 kg/m^2^, low normal: 18.5–21.9 kg/m^2^, normal: 22–23.9 kg/m^2^, overweight: 24–26.9 kg/m^2^, obese1: 27–29.9 kg/m^2^, obese2: ≥30 kg/m^2^

High BP: blood pressure ≥ 130/85 mmHg or on anti-hypertension medication

High FG: fasting glucose >100 mg/dL or on anti-diabetes medication

High waist circumference: ≥ 90 cm in men or ≥80 cm in women

Dyslipidemia: abnormal TG (≥ 150 mg/dL or on anti-hyperlipidemia medication) or abnormal HDL (< 40 mg/dL in men or <50 mg/dL in women)

Model 1: univariate Cox proportional hazards models

Model 2: adjusted for sex, age, education level, smoking status, physical activity, and drinking status

Model 3: adjusted for sex, age, education level, smoking status, physical activity, drinking status, BP, FG, WC and dyslipidemia

95% confidence interval for hazards ration in Model 3

## Discussion

The current study demonstrates the mortality risks of different body mass indices for adults at all ages in regard to individual cardio-metabolic profiles. A U-shaped relationship between all-cause mortality and BMI for subjects overall changes to an L-shaped association when the analysis is restricted to those having metabolic syndrome. For those with MetS, obesity-related excess mortality largely disappears except among the youngest (< 40 years). At the same time, blood pressure and blood glucose have a high PAR for mortality in both younger and older individuals, and are relevant for the prevention of premature death, irrespective of gender or body composition. The PARs for high blood pressure and hyperglycemia are 8.57% and 6.49%, respectively — higher than those for underweight (PAR = 5.80%), low normal weight (BMI = 18.5–22, PAR = 5.43%), and obesity (BMI > 30, PAR = 1.04%). The sample sizes in the present study are large enough to conduct subgroup analyses and explore the complex relationships between cardio-metabolic disorders, BMI, and mortality. We are also able to consider the role played by traditional cardiovascular risk factors on the health effects of BMI across the entire adult life course, which is missing in most studies of BMI and mortality.

### Underweight

Underweight is a well-recognized health risk for elders [1, 5, 6, 10, 11], in whom it may reflect a range of nutritional inadequacies, chronic energy deficiency, or sarcopenia [27]. However, the mortality risk of underweight for younger people is controversial; some studies reported that the U-shaped relationship between BMI and mortality also applies to younger adults [5, 7, 16], while others have found the risk to be lowest among younger subjects with the lowest BMI [28, 29]. In the current study, when the entire dataset was analyzed, we found that being underweight increased mortality risk by 51–74% (HR = 1.51–1.74) for those older than 40 years, but also that it was not a significant risk factor in multivariable models for those younger than 40 years. However, in the presence of MetS, being underweight was hazardous for premature mortality at almost all ages; this was particularly so for the youngest subgroup (20–39 years) with MetS, who experienced the highest mortality risk from being underweight (HR = 8.06, *p* < 0.01) (Fig. 2a). One of reasons that being underweight shifts from insignificance to high risk in the youngest group may be that the proportion with both high blood pressure and hyperglycemia escalates from only 1.1% in subjects overall to 75.6% for the underweight with MetS. The differences in the proportion with both high blood pressure and blood glucose in other age groups is also key to the link between being underweight and all-cause mortality; for example, for those aged 40–49 years, when the proportion increased from 3.9% for subjects overall to 65.5% for those with MetS, the mortality risk rose 2.8-fold (HR = 1.66 to 4.72).

### Metabolic syndrome and BMI

We found that the relative mortality risk with and without MetS varies between different age and BMI groups. In general, MetS increases the mortality rate less than 2-fold; its influence is intensified in the young underweight category (RR = 11.6 for the youngest age group with vs. without MetS), but is gradually attenuated with age. Our findings are in accord with most previous studies, which indicate that the mortality risk of MetS is principally identifiable in younger or middle-aged people [17–30], but not in the elderly [31, 32], except where there is other morbidity, such as chewing difficulty [33].

### Age

The current study also reveals that age-associated links between BMI and mortality are largely attributable to and more evident by way of 2 components of MetS: high blood pressure and hyperglycemia. We found the mortality risk for hypertension to be HR = 1.22 and 1.23 for those in the 20–39 years and >70 years groups, respectively; and for hyperglycemia, HR = 1.07 and 1.27, respectively, with the risks not attenuated by age. Rather, the prevalence of these risk factors substantially increases from <20% in the youngest group to about 60–70% in the oldest group. As a result, the PARs of high blood pressure and hyperglycemia increase with age, higher than the mortality burden presented by any deviant BMI at any age. These findings are consistent with prior studies. Thomas et al. followed a French cohort for 5 years and showed that hypertension, rather than other components of MetS, remained a significant predictor of all-cause mortality (HR = 1.3) in the multivariable models for those >65 [32]. Mozaffarian and colleagues followed an American elderly cohort in the Cardiovascular Health Study for 15 years [34] and found that the association of MetS with mortality was largely confined to those who had hypertension or abnormal glucose metabolism; for those with MetS but an absence of hypertension or hyperglycemia, the mortality risk was not significantly higher than for those without MetS.

### Strengths and limitations

The present study is notable for its large sample size and longitudinal study design. By the exclusion of individuals with severe comorbidities such as cancer, stroke, and heart disease, we were able to select a relatively healthy cohort to avoid confounding effects created by devastating illnesses [19, 35, 36]. Confidence in our findings is encouraged because of the consistency in findings on subgroup analyses and by its restriction to non-smokers, stratification by gender, and deletion of those who died within 3 years after the index date.

However, the study also has several limitations. First, the selection of the participants through a private health screening center may have induced bias toward higher socioeconomic class, so our results may not be generalizable to individuals with lower incomes. Second, because only the baseline data were used in this study, misclassifications are possible for blood pressure, blood glucose, and BMI during follow-up; however, the single determinations at baseline have enabled us to predict subsequent mortality outcomes. Third, as with other currently available studies of populations dominantly of Chinese ethnicity [8], only a small proportion were obese (3.62% with BMI ≥ 30 kg/m^2^ and 0.39% ≥ 35 kg/m^2^). We may have, therefore, underestimated the harmful effects of obesity, especially among the elderly. Fourth, as shown in previous studies, the relationship between obesity and metabolic disorders or other medical complications is complex [37–39], and the observed low obesity-related mortality may be due to medical or public health advances [40]. We were unable to fully tease out any residual effects of obesity on mortality risk that might operate through unrecognized or unmeasured metabolic disorders, although multivariable proportional hazards models were used to control for available possible confounding covariates. Finally, ours is an observational study and, although of longitudinal design, disallows ascertainment of causality.

## Conclusions

In conclusion, the effect of BMI on mortality varies with age. Obesity is associated with a higher mortality risk for younger people aged 20–49 years, while underweight incurs an excess death risk mainly for elders. For those older than 50 years, even a BMI of 18.5–21.9 kg/m^2^, usually regarded as a “normal body weight”, could increase the mortality rate by 14–25%, compared to a BMI of 22–23.9 kg/m^2^. On the other hand, high blood pressure and hyperglycemia generally impose a higher mortality burden than any deviant BMI. From the public health perspective, weight-management programs almost certainly need to be tailored according to age. However, life-extension policies warrant attention among all age groups to cardio-metabolic disorders such as hypertension, pre-diabetes, and diabetes.

## Additional file

Additional file 1: Table S1. All-cause mortality risk for subjects overall and those who survived at least 1, 2, and 3 years after entry. **Figure S1.** Adjusted mortality risk for different BMI, high blood pressure, hyperglycemia, and waist circumference for overall subjects, non-smokers, men, and women, stratified by age. BMI classification: underweight: <18.5 kg/m^2^, low normal: 18.5–21.9 kg/m^2^, normal: 22–23.9 kg/m^2^, overweight: 24–26.9 kg/m^2^, obese1: 27–29.9 kg/m^2^, obese2: ≥ 30 kg/m^2^. The hazards ratios shown in **Figure S1** were derived from Cox proportional hazards models adjusted for gender, age, education level, smoking status, physical activity, and drinking status. **Table S2.** Mortality rate (per 10,000 person-years) by body mass index, age, and status of metabolic syndrome for the study subjects including previous heart disease and stroke (*N* = 390,941). **Table S3.** Mortality risk, prevalence, and population attributable burden of mortality for different BMI, high blood pressure, and hyperglycemia in overall subjects and people in different age groups, for the study subjects including previous heart disease and stroke (*N* = 390,941). (DOCX 135 kb)

## Abbreviations

BMI: Body mass index
CI: Confidence interval
CMF: Cardio-metabolic factors
HR: Hazard ratio
LTPA: Leisure time physical activity
MetS: Metabolic syndrome
PAR: Population attributable risk
SD: Standard deviation
