# Supplementary material for: Cardiometabolic disorder reduces survival prospects more than suboptimal body mass index irrespective of age or gender: a longitudinal study of 377,929 adults in Taiwan
Source: BMC Public Health. 2018 Feb 14;18:142. doi: 10.1186/s12889-018-5038-0 (PMC5812051; doi:10.1186/s12889-018-5038-0)
Supplement: Supplementary file 1 — All-cause mortality risk for subjects overall and those who survived at least 1, 2, and 3 years after entry. Figure S1. Adjusted mortality risk for different BMI, high blood pressure, hyperglycemia, and waist circumference for overall subjects, non-smokers, men, and women, stratified by age. BMI classification: underweight: <18.5 kg/m2, low normal: 18.5–21.9 kg/m2, normal: 22–23.9 kg/m2, overweight: 24–26.9 kg/m2, obese1: 27–29.9 kg/m2, obese2: ≥ 30 kg/m2. The hazards ratios shown in Figure S1 were derived from Cox proportional hazards models adjusted for gender, age, education level, smoking status, physical activity, and drinking status. Table S2. Mortality rate (per 10,000 person-years) by body mass index, age, and status of metabolic syndrome for the study subjects including previous heart disease and stroke (N = 390,941). Table S3. Mortality risk, prevalence, and population attributable burden of mortality for different BMI, high blood pressure, and hyperglycemia in overall subjects and people in different age groups, for the study subjects including previous heart disease and stroke (N = 390,941). (DOCX 135 kb) [file 12889_2018_5038_MOESM1_ESM.docx]

**Additional files - Table and Figure**

Table S1 All-cause mortality risk for subjects overall and those who survived at least 1, 2, and 3 years after entry

| Hazard Ratio (95% Confidence Interval) | | | | | | | |
| --- | --- | --- | --- | --- | --- | --- | --- |
| Age | n | underweight | low normal | Normal | overweight | obese1 | obese2 |
|  |  | **Overall** | | | | | |
| 20-39 | 208986 | 1.1 (0.9-1.5) | 1.0 (0.8-1.2) | 1 | 1.0 (0.8-1.2) | 1.4 (1.0-1.8)* | 2.6 (2.0-3.4)** |
| 40-49 | 70021 | 1.6 (1.1-2.3)** | 1.1 (0.9-1.3) | 1 | 1.0 (0.9-1.3) | 1.3 (1.0-1.6)* | 1.8 (1.4-2.4)** |
| 50-59 | 54952 | 1.7 (1.4-2.2)** | 1.1 (0.9-1.2) | 1 | 0.9 (0.8-1.1) | 1.2 (1.0-1.3)* | 1.4 (1.1-1.7)** |
| 60-69 | 32594 | 1.5 (1.3-1.8)** | 1.1 (1.0-1.2) | 1 | 1.0 (0.9-1.0) | 1.1 (1.0-1.2) | 1.3 (1.1-1.5)** |
| ≥70 | 10811 | 1.6 (1.3-1.8)** | 1.2 (1.1-1.3)** | 1 | 1.0 (0.9-1.1) | 1.0 (0.9-1.2) | 1.2 (1.0-1.5) |
|  |  | **Delete those who died within 1 year after index date** | | | | | |
| 20-39 | 208943 | 1.1 (0.8-1.4) | 1.0 (0.8-1.2) | 1 | 1.0 (0.8-1.2) | 1.4 (1.1-1.8)** | 2.7 (2.0-3.5)** |
| 40-49 | 69993 | 1.6 (1.1-2.3)** | 1.1 (0.9-1.3) | 1 | 1.1 (0.9-1.3) | 1.3 (1.0-1.6)* | 1.8 (1.4-2.4)** |
| 50-59 | 54885 | 1.6 (1.3-2.1)** | 1.1 (0.9-1.2) | 1 | 0.9 (0.8-1.1) | 1.1 (1.0-1.3) | 1.4 (1.1-1.7)** |
| 60-69 | 32509 | 1·5 (1.2-1.8)** | 1.1 (1.0-1.2) | 1 | 1.0 (0.9-1.1) | 1.1 (1.0-1.2) | 1.3 (1.1-1.5)** |
| ≥70 | 10736 | 1·6 (1.3-1.8)** | 1.2 (1.1-1.3)** | 1 | 1.0 (0.9-1.1) | 1.0 (0.9-1.2) | 1.2 (1.0-1.5) |
|  |  | **Delete those who died within 2 years after index date** | | | | | |
| 20-39 | 208874 | 1.1 (0.8-1.4) | 0.9 (0.8-1.1) | 1 | 1.0 (0.8-1.2) | 1.4 (1.1-1.8)* | 2.8 (2.1-3.7)** |
| 40-49 | 69930 | 1.8 (1.3-2.6)** | 1.1 (0.9-1.4) | 1 | 1.1 (0.9-1.3) | 1.3 (1.0-1.7)* | 1.9 (1.4-2.5)** |
| 50-59 | 54759 | 1.6 (1.3-2.2)** | 1.1 (0.9-1.2) | 1 | 1.0 (0.8-1.1) | 1.1 (1.0-1.3) | 1.3 (1.1-1.7)** |
| 60-69 | 32281 | 1.4 (1.1-1.6)** | 1.1 (1.0-1.2) | 1 | 1.0 (0.9-1.1) | 1.1 (0.9-1.2) | 1.3 (1.1-1.5)** |
| ≥70 | 10581 | 1.5 (1.3-1.8)** | 1.2 (1.0-1.3)** | 1 | 1.0 (0.9-1.1) | 1.0 (0.9-1.2) | 1.2 (1.0-1.5) |
|  |  | **Delete those who died within 3 years after index date** | | | | | |
| 20-39 | 208795 | 1.0 (0.8-1.4) | 0.9 (0.8-1.2) | 1 | 1.0 (0.8-1.3) | 1.5 (1.1-2.0)** | 2.8 (2.1-3.8)** |
| 40-49 | 69850 | 1.8 (1.2-2.6)** | 1.1 (0.9-1.4) | 1 | 1.1 (0.9-1.3) | 1.3 (1.0-1.7)* | 1.8 (1.4-2.5)** |
| 50-59 | 54615 | 1.6 (1.2-2.2)** | 1.1 (0.9-1.3) | 1 | 1.0 (0.8-1.1) | 1.1 (1.0-1.3) | 1.4 (1.1-1.7)** |
| 60-69 | 32047 | 1.4 (1.2-1.7)** | 1.1 (1.0-1.2) | 1 | 1.0 (0.9-1.1) | 1.1 (0.9-1.2) | 1.2 (1.0-1.5)* |
| ≥70 | 10370 | 1.5 (1.3-1.8)** | 1.2 (1.0-1.3)* | 1 | 1.0 (0.9-1.1) | 1.1 (0.9-1.2) | 1.2 (1.0-1.5) |

Multivariate model adjusted for sex, age, education status, smoking status, drinking status, and physical activity

*P<0.05, **P<0.01.

Figure S1 Adjusted mortality risk for different BMI, high blood pressure, hyperglycemia, and waist circumference for overall subjects, non-smokers, men, and women, stratified by age


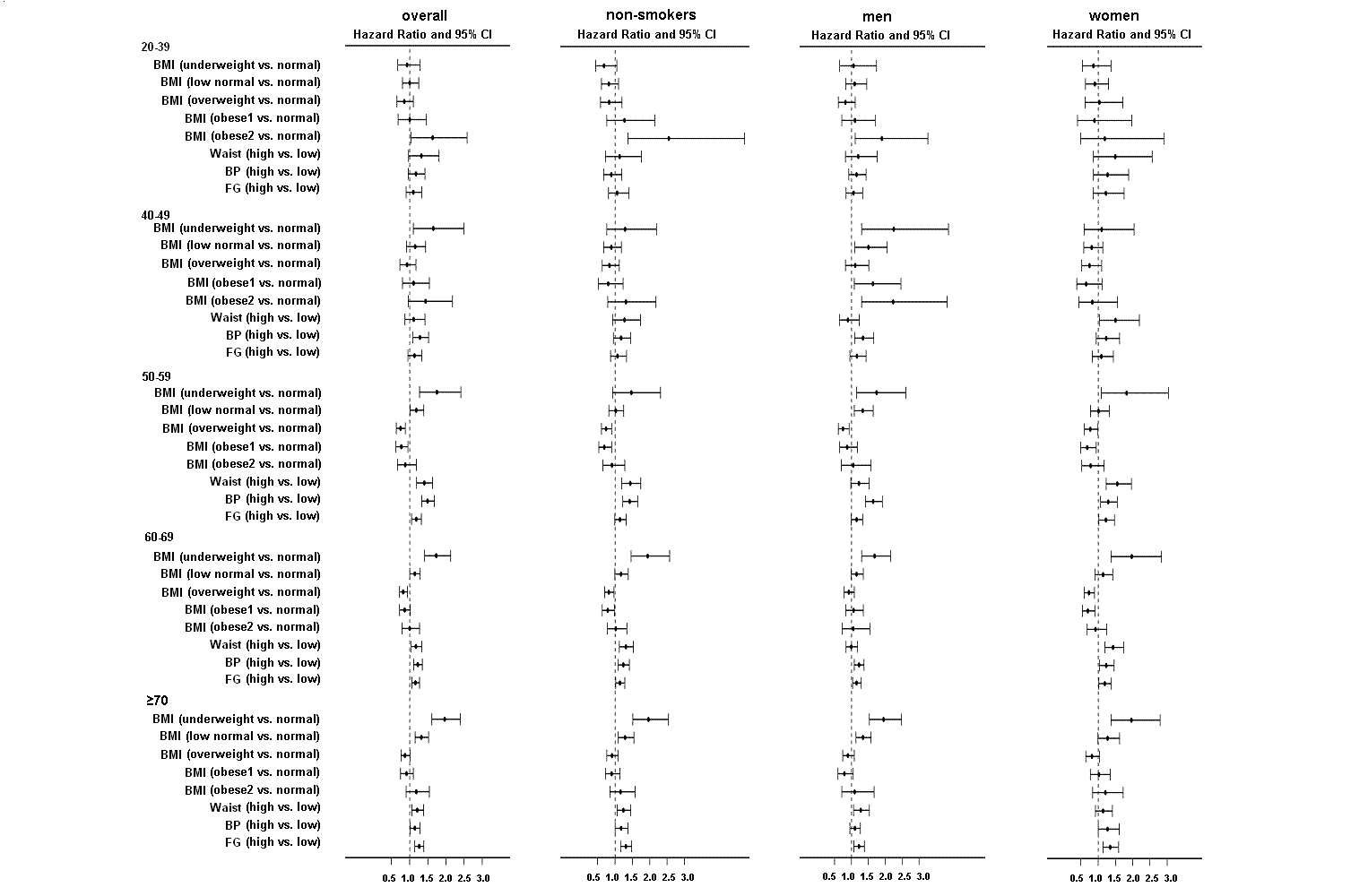


Table S2 Mortality rate (per 10,000 person-years) by body mass index, age, and status of metabolic syndrome for the study subjects including previous heart disease and stroke (N=390,941)

|  | underweight | | | | Low normal | | | | normal | | | | overweight | | | | obese1 | | | | obese2 | | | | |
| --- | --- | --- | --- | --- | --- | --- | --- | --- | --- | --- | --- | --- | --- | --- | --- | --- | --- | --- | --- | --- | --- | --- | --- | --- | --- |
|  | No. of deaths | person years (× 10^3^) | Death rate | RR | No. of deaths | person years (× 10^3^) | Death rate | RR | No. of deaths | person years (× 10^3^) | Death rate | RR | No. of deaths | person years (×10^3^) | Death rate | RR | No. of deaths | person years (× 10^3^) | Death rate | RR | No. of deaths | person years (× 10^3^) | Death rate | RR | |
| **Overall** |  |  |  |  |  |  |  |  |  |  |  |  |  |  |  |  |  |  |  |  |  |  |  |  | |
| **20-39** | 98 | 204.6 | 4.8 |  | 325 | 690.9 | **4.7** |  | 183 | 327.6 | 5.6 |  | 174 | 279.8 | 6.2 |  | 85 | 98.4 | 8.6 |  | 69 | 43.9 | 15.7 |  | |
| **40-49** | 38 | 19.0 | 20.0 |  | 224 | 162.4 | **13.8** |  | 212 | 146.4 | 14.5 |  | 275 | 166.7 | 16.5 |  | 139 | 64.2 | 21.7 |  | 77 | 24.5 | 31.4 |  | |
| **50-59** | 79 | 9.9 | 79.8 |  | 456 | 97.9 | 46.6 |  | 529 | 119.9 | 44.1 |  | 650 | 156.1 | **41.6** |  | 331 | 66.0 | 50.2 |  | 133 | 24.3 | 54.7 |  | |
| **60-69** | 181 | 9.4 | 192.6 |  | 816 | 64.3 | 126.9 |  | 872 | 76.1 | 114.6 |  | 1110 | 102.0 | **108.8** |  | 504 | 42.5 | 118.6 |  | 200 | 15.4 | 129.9 |  | |
| **≥70** | 273 | 5.1 | 535.3 |  | 858 | 23.2 | 369.8 |  | 718 | 24.0 | 299.2 |  | 878 | 31.9 | 275.2 |  | 357 | 13.1 | **272.5** |  | 134 | 4.3 | 311.6 |  | |
| **With MetS** |  |  |  |  |  |  |  |  |  |  |  |  |  |  |  |  |  |  |  |  |  |  |  |  | |
| **20-39** | 4 | 0.8 | 50.0 | 11.0  (0.2-20.7) | 13 | 11.0 | 11.8 | 2.6  (1.2-4.0) | 15 | 19.4 | **7.7** | 1.5  (0.7-2.2) | 45 | 45.6 | 9.9 | 1.8  (1.3-2.4) | 37 | 36.9 | 10.0 | 1.5  (1.0-2.0) | 42 | 25.2 | 16.7 | 1.4  (0.9-1.8) | |
| **40-49** | 3 | 0.3 | 100.0 | 5.4  (0-12.7) | 25 | 7.4 | 33.8 | 2.7  (1.6-3.7) | 36 | 17.2 | **20.9** | 1.6  (1.0-2.1) | 94 | 44.7 | 21.0 | 1.5  (1.2-1.8) | 83 | 31.1 | 26.7 | 1.4  (1.1-1.7) | 52 | 15.8 | 32.9 | **1.5**  **(1.1-1.9)** | |
| **50-59** | 4 | 0.3 | 133.3 | 1.8  (0-3.2) | 85 | 10.6 | 80.2 | 2.0  (1.6-2.4) | 162 | 26.4 | 61.4 | 1.7  (1.4-1.9) | 288 | 58.7 | **49.1** | 1.4  (1.3-1.6) | 215 | 38.4 | 56.0 | 1.5  (1.3-1.7) | 89 | 16.6 | 53.6 | 1.0  (0.8-1.2) | |
| **60-69** | 19 | 0.6 | 316.7 | 1.7  (0.9-2.4) | 194 | 11.8 | 164.4 | 1.4  (1.2-1.7) | 332 | 23.9 | 138.9 | 1.4  (1.2-1.5) | 596 | 51.1 | **116.6** | 1.2  (1.1-1.3) | 366 | 28.2 | 129.8 | 1.4  (1.2-1.5) | 147 | 11.5 | 127.8 | 1.1  (0.9-1.2) | |
| **≥70** | 38 | 0.4 | 950.0 | 2.0  (1.3-2.5) | 272 | 5.8 | 469.0 | 1.5  (1.3-1.7) | 328 | 9.9 | 331.3 | 1.3  (1.2-1.5) | 530 | 18.2 | 291.2 | 1.3  (1.2-1.5) | 269 | 9.4 | **286.2** | 1.7  (1.5-1.9) | 105 | 3.2 | 328.1 | 1.3  (1.0-1.4) | |
| **Without MetS** |  |  |  |  |  |  |  |  |  |  |  |  |  |  |  |  |  |  |  |  |  |  |  |  |  |
| **20-39** | 91 | 200.9 | **4.5** |  | 299 | 659.7 | **4.5** |  | 153 | 291.2 | 5.3 |  | 115 | 213.9 | 5.4 |  | 36 | 53.2 | 6.8 |  | 19 | 15.4 | 12.3 |  |  |
| **40-49** | 34 | 18.4 | 18.5 |  | 189 | 148.6 | **12.7** |  | 161 | 119.7 | 13.5 |  | 145 | 105.9 | 13.7 |  | 50 | 26.8 | 18.7 |  | 14 | 6.2 | 22.6 |  |  |
| **50-59** | 68 | 9.2 | 73.9 |  | 332 | 81.4 | 40.8 |  | 309 | 83.6 | 37.0 |  | 282 | 82.6 | **34.1** |  | 79 | 21.2 | 37.3 |  | 30 | 5.4 | 55.6 |  |  |
| **60-69** | 146 | 8.0 | 182.5 |  | 538 | 47.4 | 113.5 |  | 443 | 44.5 | 99.6 |  | 386 | 40.5 | **95.3** |  | 99 | 10.3 | 96.1 |  | 31 | 2.6 | 119.2 |  |  |
| **≥70** | 210 | 4.4 | 477.3 |  | 483 | 15.6 | 309.6 |  | 301 | 11.9 | 252.9 |  | 233 | 10.7 | 217.8 |  | 41 | 2.4 | **170.8** |  | 15 | 0.6 | 250.0 |  |  |

MetS: metabolic syndrome

RR: mortality rate ratio, comparing the mortality rate for individuals with MetS to those without MetS, with regard to the corresponding BMI-age subgroups.

The numbers in bold indicate the lowest death rate in the respective age groups.

Table S3 Mortality risk, prevalence, and population attributable burden of mortality for different BMI, high blood pressure, and hyperglycemia in overall subjects and people in different age groups, for the study subjects including previous heart disease and stroke (N=390,941)

|  | Model 1  HR | Model 2  HR | Model 3  HR | 95% CI | Prevalence (%) | PAR (%) |
| --- | --- | --- | --- | --- | --- | --- |
| **Overall (n=390,941 , death=10978)** |  |  |  |  |  |  |
| Underweight (vs. normal BMI) | **0**.**77***** | **1**.**53***** | **1**.7**3***** | (1.55–1.92) | 8.32 | 5.72 |
| Low normal (vs. normal BMI) | **0**.**72***** | **1**.**09***** | **1**.**18***** | (1.11–1.27) | 33.37 | 5.67 |
| Normal BMI | 1 | 1 | 1 | - | 22.08 | - |
| Overweight (vs. normal BMI) | **1**.**16***** | 0.96 | **0**.**82***** | (0.76–0.88) | 23.41 | -4.40 |
| Obese1 (vs. normal BMI) | **1**.**37***** | **1**.**09**** | **0.85***** | (0.78–0.94) | 9.09 | -1.38 |
| Obese2 (vs. normal BMI) | **1**.**52***** | **1**.**41***** | 1.06 | (0.94–1.20) | 3.73 | 0.22 |
| High BP (vs. normal BP) | **4**.**12***** | **1**.**35***** | **1**.**30***** | (1.23–1.37) | 31.30 | 8.58 |
| High FG (vs. normal FG) | **2**.**60***** | **1**.**25***** | **1**.**17***** | (1.12–1.23) | 30.78 | 4.97 |
| High WC (vs. normal WC) | **2**.**53***** | **1**.**12***** | **1**.**23***** | (1.15–1.31) | 19.12 | 4.21 |
| Dyslipidemia vs. normal TG/HDL | **1**.**40***** | **1**.**14***** | **1**.**17***** | (1.11–1.23) | 43.33 | 6.86 |
|  |  |  |  |  |  |  |
| **20-39 (n= 211,590, death=934)** |  |  |  |  |  |  |
| Underweight (vs. normal BMI) | 0.87 | 1.16 | 1.00 | (0.73–1.37) | 12.70 | 0 |
| Low normal (vs. normal BMI) | 0.84 | 0.99 | 1.02 | (0.82–1.27) | 41.21 | 0.82 |
| Normal BMI | 1 | 1 | 1 | - | 19.78 | - |
| Overweight (vs. normal BMI) | 1.12 | 0.99 | 0.85 | (0.65–1.10) | 17.18 | -2.65 |
| Obese1 (vs. normal BMI) | **1**.**56***** | **1**.**35*** | 1.01 | (0.69–1.47) | 6.20 | 0.06 |
| Obese2 (vs. normal BMI) | **2**.**89***** | **2**.**63***** | **1.65*** | (1.05–2.59) | 2.92 | 1.86 |
| High BP (vs. normal BP) | **1**.**57***** | **1**.**37***** | 1.18 | (0.98–1.43) | 16.88 | 2.95 |
| High FG (vs. normal FG) | **1**.**44***** | **1**.**20*** | 1.14 | (0.94–1.38) | 19.15 | 2.61 |
| High WC (vs. normal WC) | **2**.**07***** | **1**.**68***** | 1.31 | (0.96–1.79) | 10.20 | 3.07 |
| Dyslipidemia vs. normal TG/HDL | **1**.**37***** | **1**.**22**** | **1**.**20*** | (1.01–1.41) | 37.92 | 7.05 |
|  |  |  |  |  |  |  |
| **40-49 (n= 71,763, death=965)** |  |  |  |  |  |  |
| Underweight (vs. normal BMI) | **1**.**43*** | **1**.**59**** | **1**.**67*** | (1.12–2.50) | 3.55 | 2.33 |
| Low normal (vs. normal BMI) | 0.97 | 1.09 | 1.16 | (0.92–1.45) | 28.45 | 4.35 |
| Normal BMI | 1 | 1 | 1 | - | 24.73 | - |
| Overweight (vs. normal BMI) | 1.14 | 1.04 | 0.93 | (0.74–1.17) | 28.19 | -2.01 |
| Obese1 (vs. normal BMI) | **1**.**50***** | **1**.**31*** | 1.17 | (0.86–1.61) | 10.90 | 1.82 |
| Obese2 (vs. normal BMI) | **2**.**17***** | **1**.**90***** | **1**.**52*** | (1.02–2.26) | 4.16 | 2.12 |
| High BP (vs. normal BP) | **1**.**63***** | **1**.**45***** | **1**.**29**** | (1.09–1.51) | 30.79 | 8.20 |
| High FG (vs. normal FG) | **1**.**45***** | **1**.**28***** | 1.13 | (0.96–1.33) | 35.52 | 4.41 |
| High WC (vs. normal WC) | **1**.**43***** | **1**.**22*** | 1.07 | (0.84–1.36) | 20.65 | 1.42 |
| Dyslipidemia vs. normal TG/HDL | **1**.**21**** | 1.07 | 1.00 | (0.85–1.18) | 47.00 | 0 |
|  |  |  |  |  |  |  |
| **50-59 (n= 58,075, death=2178)** |  |  |  |  |  |  |
| Underweight (vs. normal BMI) | **1**.**86***** | **1**.**77***** | **1**.**90***** | (1.42–2.56) | 2.18 | 1.93 |
| Low normal (vs. normal BMI) | 1.07 | 1.06 | **1**.**17*** | (1.00–1.37) | 21.05 | 3.45 |
| Normal BMI | 1 | 1 | 1 | - | 25.24 | - |
| Overweight (vs. normal BMI) | 0.94 | 0.93 | **0**.**74***** | (0.63–0.86) | 32.63 | -9.27 |
| Obese1 (vs. normal BMI) | 1.13 | 1.12 | **0**.**73**** | (0.59–0.89) | 13.72 | -3.85 |
| Obese2 (vs. normal BMI) | **1**.**24*** | **1**.**30**** | 0.83 | (0.63–1.08) | 5.18 | -0.89 |
| High BP (vs. normal BP) | **1**.**50***** | **1**.**50***** | **1**.**54***** | (1.37–1.72) | 51.15 | 21.64 |
| High FG (vs. normal FG) | **1**.**38***** | **1**.**33***** | **1**.**16**** | (1.04–1.29) | 47.28 | 7.03 |
| High WC (vs. normal WC) | **1**.**23***** | **1**.**22***** | **1**.**40***** | (1.21–1.63) | 32.14 | 11.39 |
| Dyslipidemia vs. normal TG/HDL | **1**.**15**** | **1**.**10*** | 1.09 | (0.98–1.22) | 49.97 | 4.30 |
|  |  |  |  |  |  |  |
| **60-69 (n= 36,451, death=3683)** |  |  |  |  |  |  |
| Underweight (vs. normal BMI) | **1**.**68***** | **1**.**44***** | **1**.**68***** | (1.38–2.05) | 3.03 | 2.02 |
| Low normal (vs. normal BMI) | **1**.**11*** | 1.06 | 1.12 | (0.99–1.26) | 20.83 | 2.44 |
| Normal BMI | 1 | 1 | 1 | - | 24.65 | - |
| Overweight (vs. normal BMI) | 0.95 | 0.96 | **0**.**83**** | (0.73–0.93) | 32.73 | -5.89 |
| Obese1 (vs. normal BMI) | 1.03 | 1.08 | 0.86 | (0.73–1.01) | 13.66 | -1.95 |
| Obese2 (vs. normal BMI) | 1.14 | **1**.**27**** | 0.98 | (0.79–1.21) | 5.10 | -0.10 |
| High BP (vs. normal BP) | **1**.**22***** | **1**.**27***** | **1**.**24***** | (1.14–1.37) | 67.19 | 13.89 |
| High FG (vs. normal FG) | **1**.**21***** | **1**.**24***** | **1**.**18***** | (1.08–1.28) | 53.26 | 8.75 |
| High WC (vs. normal WC) | 0.97 | 1.08 | **1**.**17**** | (1.05–1.31) | 40.51 | 6.44 |
| Dyslipidemia vs. normal TG/HDL | **1**.**14***** | **1**.**17***** | **1**.**17***** | (1.07–1.28) | 53.43 | 8.33 |
|  |  |  |  |  |  |  |
| **≥70 (n= 13,062, death=3218)** |  |  |  |  |  |  |
| Underweight (vs. normal BMI) | **1**.**80***** | **1**.**57***** | **1**.**94***** | (1.63–2.32) | 5.47 | 4.89 |
| Low normal (vs. normal BMI) | **1**.**24***** | **1**.**16**** | **1**.**29***** | (1.14–1.46) | 23.08 | 6.27 |
| Normal BMI | 1 | 1 | 1 | - | 23.64 | - |
| Overweight (vs. normal BMI) | 0.91 | 0.93 | **0**.**85*** | (0.74–0.96) | 30.99 | -4.88 |
| Obese1 (vs. normal BMI) | 0.90 | 0.95 | **0**.**84*** | (0.70–0.99) | 12.61 | -2.06 |
| Obese2 (vs. normal BMI) | 1.04 | 1.17 | 1.05 | (0.82–1.33) | 4.21 | 0.21 |
| High BP (vs. normal BP) | **1**.**21***** | **1**.**26***** | **1**.**19**** | (1.06–1.33) | 79.15 | 13.07 |
| High FG (vs. normal FG) | **1**.**19***** | **1**.**22***** | **1**.**20***** | (1.10–1.31) | 57.12 | 10.25 |
| High WC (vs. normal WC) | **0**.**89**** | 0.97 | **1**.**17**** | (1.04–1.32) | 46.39 | 7.31 |
| Dyslipidemia vs. normal TG/HDL | 1.07 | **1**.**13**** | **1**.**25***** | (1.14–1.36) | 52.96 | 11.69 |

*P<0.05, **P<0.01, ***P<0.001.

BMI: body mass index, BP: blood pressure, FG: fasting glucose level, WC: waist circumference, TG: triglyceride, HDL: high density lipoprotein cholesterol

BMI classification: Underweight: <18.5 kg/m^2^, low normal: 18.5-21.9 kg/m^2^, normal: 22-23.9 kg/m^2^, overweight: 24-26.9 kg/m^2^, obese1: 27-29.9 kg/m^2^, obese2: ≥30 kg/m^2^

High BP: blood pressure ≥ 130/85 mmHg or on anti-hypertension medication

High FG: fasting glucose > 100 mg/dL or on anti-diabetes medication

High waist circumference: ≥ 90 cm in men or ≥ 80 cm in women

Dyslipidemia: abnormal TG (≥ 150 mg/dL or on anti-hyperlipidemia medication) or abnormal HDL (< 40 mg/dL in men or < 50 mg/dL in women)

Model 1: univariate Cox proportional hazards models

Model 2: adjusted for sex, age, education level, smoking status, physical activity, and drinking status.

Model 3: adjusted for sex, age, education level, smoking status, physical activity, drinking status, BP, FG, WC and Dyslipidemia.
